# Perioperative outcomes of combined heart surgery and lung tumor resection: a systematic review and meta-analysis

**DOI:** 10.1186/s13019-021-01607-7

**Published:** 2021-08-09

**Authors:** Shizhao Cheng, Yiyao Jiang, Xin Li, Xike Lu, Xun Zhang, Daqiang Sun

**Affiliations:** 1 Department of Thoracic Surgery, Tianjin Chest Hospital, Tianjin, 300222 China; 2 Department of Cardiac Surgery, Tianjin First Central Hospital, Tianjin, 300074 China

**Keywords:** Heart surgery, Lung surgery, Perioperative outcomes, Combined cardiothoracic surgery, Meta-analysis

## Abstract

**Objective:**

The prevalence of patients with concomitant heart and lung lesions requiring surgical intervention is increasing. Simultaneous cardiac surgery and pulmonary resection avoids the need for a second operation. However, there are concerns regarding the potentially increased mortality and complication rates of simultaneous surgery and the adequacy of lung exposure during heart surgery. Therefore, we performed a meta-analysis to evaluate the perioperative mortality and complication rates of combined heart surgery and lung tumor resection.

**Methods:**

A comprehensive literature search was performed in July 2020. The PubMed, Embase, and Web of Science databases were searched to identify studies that reported the perioperative outcomes of combined heart surgery and lung tumor resection. Two reviewers independently screened the studies, extracted data, and assessed the risk of bias of included studies. Pooled proportions and 95% confidence intervals (95% CI) were calculated by R version 3.6.1 using the meta package.

**Results:**

A total of 536 patients from 29 studies were included. Overall, the pooled proportion of operative mortality was 0.01 (95% CI: 0.00, 0.03) and the pooled proportion of postoperative complications was 0.40 (95% CI: 0.24, 0.57) for patients who underwent combined cardiothoracic surgery. Subgroup analysis by lung pathology revealed that, for patients with lung cancer, the pooled proportion of anatomical lung resection was 0.99 (95% CI: 0.95, 1.00) and the pooled proportion of systematic lymph node dissection or sampling was 1.00 (95% CI: 1.00, 1.00). Subgroup analysis by heart surgery procedure found that the pooled proportion of postoperative complications of patients who underwent coronary artery bypass grafting (CABG) patients using the off-pump method was 0.17 (95% CI: 0.01, 0.43), while the pooled proportion of complications after CABG using the on-pump method was 0.61 (95% CI: 0.38, 0.82).

**Conclusion:**

Combined heart surgery and lung tumor resection had a low mortality rate and an acceptable complication rate. Subgroup analyses revealed that most patients with lung cancer underwent uncompromised anatomical resection and mediastinal lymph node sampling or dissection during combined cardiothoracic surgery, and showed off-pump CABG may reduce the complication rate compared with on-pump CABG. Further researches are still needed to verify these findings.

## Introduction

Although relatively few patients have concomitant lesions of the heart and lungs requiring surgical intervention, the prevalence of such patients is increasing [1–3]. Some patients, who required cardiac surgery, found asymptomatic indeterminate lung nodules during preoperative examination. Other patients, who were scheduled to receive lung tumor resection, had heart diseases requiring surgical intervention. The management of patients who require both heart surgery and lung resection is challenging. Treatment options include either simultaneous or staged surgical procedures [4–8]. Without postponing the treatment of either heart or lung diseases, simultaneous surgery can solve heart and lung lesions at the same time, thus avoiding the second operation [3, 9–12]. However, there are concerns regarding the potentially increased mortality and complication rates of simultaneous surgery and the adequacy of lung exposure during heart surgery [8, 12–20].

To address these concerns, we conducted this meta-analysis to systematically evaluate the perioperative mortality and complication rates of combined heart surgery and lung tumor resection. In the subgroup analyses, we also investigated the proportion of patients with lung cancer who underwent anatomic lung resection and mediastinal lymph node sampling or dissection, and the impact of cardiopulmonary bypass (CPB) on perioperative complications.

## Methods

### Search strategy

**Fig. 1:**
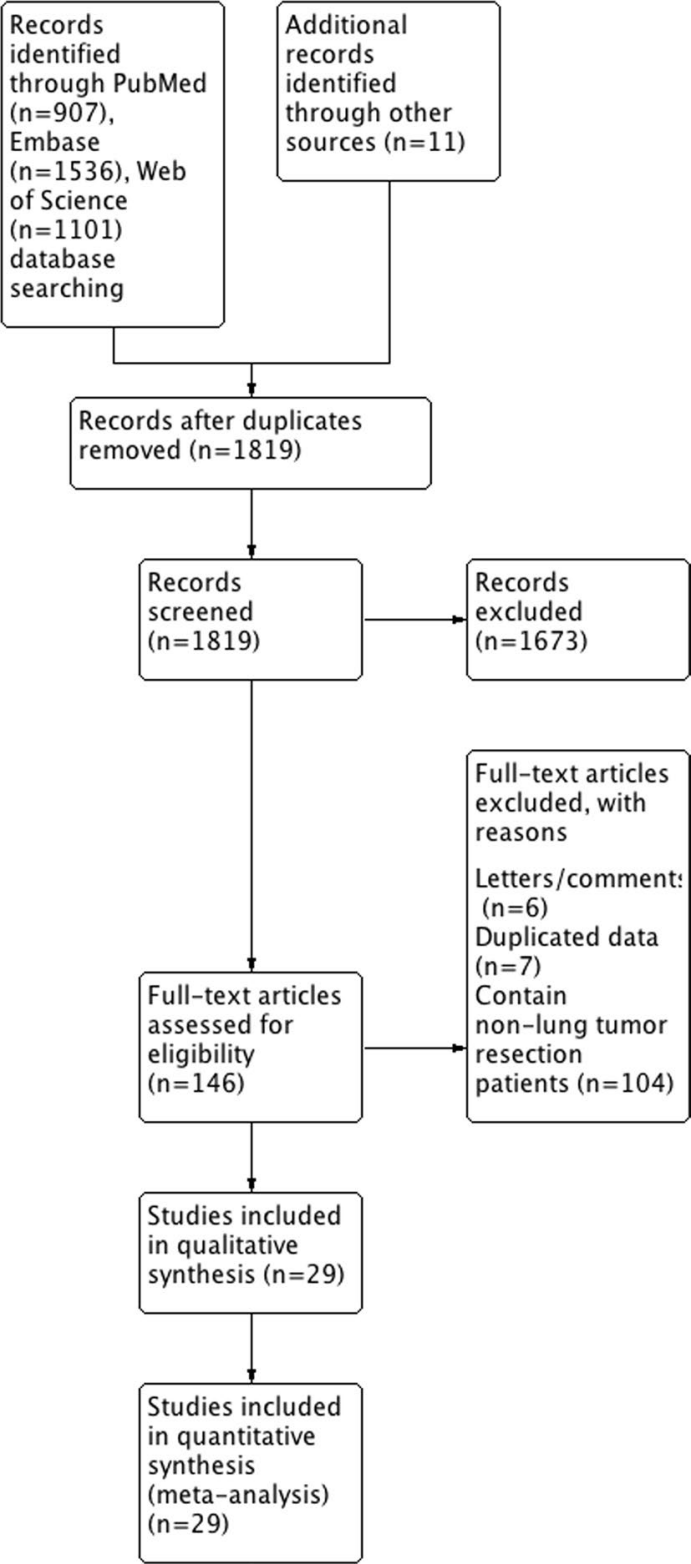
Flow diagram of included studies for this meta-analysis

A comprehensive literature search was conducted using PubMed, Embase and Web of Science database in 7 July 2020 to identify citations reporting perioperative outcomes of combined heart surgery and lung tumor resection. The following search algorithm was used: (exp Cardiac Surgical Procedures/ or ((heart$ or cardio$ or cardiac or coronary or myocardial or valve$) adj1 (surger$ or operation$ or graft$ or bypass$ or revascularization$ or plasty or replacement)).tw.) and (exp Pneumonectomy/ or ((lung$ or pulmon$ or sublob$ or wedge$) adj1 (surger$ or operation$ or resect$)).tw.). In addition, the reference lists of the retrieved articles and related reviews were checked to identify potentially relevant additional studies. No language or publication date restrictions were adopted. The flow diagram was presented in Fig. 1. The present meta-analysis was designed, performed, and reported in accordance with the standards of quality for reporting meta-analysis.

### Selection criteria

Articles included in this study had to meet all the following criteria: the exposure of interest was combined heart surgery and lung tumor resection whether benign or malignant; the outcome of interest was the rates of operative mortality and postoperative complications; the study design was a case–control, retrospective, or prospective study; and the proportion estimates with their corresponding 95% confidence intervals (95% CI) were reported or sufficient data were provided to calculate them. Operative mortality was defined as death within 30 days of operation or during hospitalization. Postoperative complications were defined as the occurrence of complications within 30 days of operation or during the postoperative stay.

The included studies focused on lung tumor resection. Publications were excluded if they involved lung transplantation surgery, trauma surgery, lung infectious disease surgery, esophageal surgery, or mediastinal surgery. Reviews, editorial commentaries, or studies with no extractable data were also excluded.

### Quality assessment

Newcastle–Ottawa Scale (NOS) was used to evaluate the quality of included researches by two independent reviewers (Yiyao Jiang, Xin Li). NOS is a 9-star system, which includes 3 dimensions: selection (4 items), comparability (1 item), and exposure/outcome (3 items). Each item represents 1 point, except for comparability (2 points). A study with ≥ 7 points was considered as a high-quality study.

### Data extraction

Data were extracted independently by the two reviewers (Shizhao Cheng, Yiyao Jiang) and any discrepancies were resolved by discussion. The following data were extracted from each included study: publication details, sample size, lung tumor histological type, and operative mortality and postoperative complication proportion estimates with their corresponding 95% CI.

### Statistical analysis

All quantitative analysis was performed in R version 3.6.1 using the meta package. For the meta-analysis of the operative mortality and postoperative complications proportions, both fixed-effect and random-effect approaches were adopted using the Freeman-Tukey double arcsine transformation method [21]. Subgroup analyses were performed based on different lung pathology or heart surgery procedures. Heterogeneity across included studies was assessed with the Cochran Q test (the level of significance was set at 0.1) [22]. The I^2^ score was used to determine the degree of heterogeneity (I^2^ < 50%, no obvious heterogeneity; I^2^ > 50%, large or extreme heterogeneity). In sensitivity analysis, meta-analysis was conducted after omitting each study in turn. Potential publication bias was evaluated using Begg’s test and Egger’s test [23].

## Results

### Literature search and study characteristics

The detailed process of literature retrieval procedures is shown in Fig. 1. A total of 29 eligible studies were finally included in the present meta-analysis [3, 5–20, 24–35]. These studies were carried out in the following geographical regions: five in the USA [8, 19, 20, 32, 35], four in China [3, 11, 25, 26], four in the Netherlands [7, 13, 30, 33], three in Italy [14, 18, 29], two in the Czech Republic [12, 24], two in Japan [10, 16], two in Turkey [9, 15], one in Australia [34], one in Belgium [6], one in Canada [31], one in Germany [5], one in Greece [27], one in Poland [28], and one in the UK [17]. All included studies were retrospective observational studies, which were published between 1990 and 2019. Of these studies, the sample sizes ranged from 2 to 79 patients. Therefore, 29 studies containing 536 patients who received combined heart surgery and lung tumor resection were subjected to final analysis. In each study, the proportion estimates with their corresponding 95% CIs of the operative mortality and postoperative complications were calculated from available information from original article. Quality scores evaluated by the Newcastle–Ottawa Scale (NOS) ranged from 5 to 7. The main characteristics of all included studies have been summarized in Table 1.

**Table 1:** Characteristics of studies included in this meta-analysis

Study	Year	Country	Study design	Study period	No. of patients	Lung pathology (Adenocarcinoma/squamous cell carcinoma/other malignant tumors/benign tumors)	Heart surgery (CABG/other types of heart surgery)	Operative mortality proportions (95% CI)	Postoperative complication proportions (95% CI)
Li	2019	China	Case–control	2009–2016	20	13/6/1/0	20/0	0.00 [0.00; 0.17]	0.00 [0.00; 0.17]
Yeginsu	2018	Turkey	Retrospective	2014–2018	10	3/6/1/0	10/0	0.10 [0.00; 0.45]	0.40 [0.12; 0.74]
Kaku	2017	Japan	Retrospective	2008–2013	18	13/2/0/3	6/12	0.00 [0.00; 0.19]	0.00 [0.00; 0.19]
Ma	2016	China	Retrospective	2003–2014	34	18/16/0/0	34/0	0.00 [0.00; 0.10]	0.32 [0.17; 0.51]
Santavy	2015	Czech Republic	Retrospective	2010–2014	10	3/0/3/4	6/4	0.00 [0.00; 0.31]	0.10 [0.00; 0.45]
Kovacicova	2014	Czech Republic	Case–control	2002–2011	12	1/7/4/0	9/3	0.00 [0.00; 0.26]	0.75 [0.43; 0.95]
Zhang	2012	Germany	Retrospective	1999–2007	30	6/5/1/18	17/13	0.07 [0.01; 0.22]	0.10 [0.02; 0.27]
Li	2012	China	Retrospective	2008–2010	3	1/2/0/0	3/0	0.00 [0.00; 0.71]	0.00 [0.00; 0.71]
Zhang	2009	China	Retrospective	2003–2008	7	2/5/0/0	7/0	0.00 [0.00; 0.41]	0.14 [0.00; 0.58]
Cathenis	2009	Belgium	Retrospective	2000–2008	27	10/14/2/1	22/5	0.00 [0.00; 0.13]	1.00 [0.87; 1.00]
Prokakis	2008	Greece	Retrospective	2004–2006	5	1/2/0/2	1/4	0.00 [0.00; 0.52]	0.40 [0.05; 0.85]
Dyszkiewicz	2008	Poland	Retrospective	2001–2006	25	6/14/5/0	25/0	0.00 [0.00; 0.14]	0.64 [0.43; 0.82]
Schoenmakers	2007	Netherlands	Case–control	1994–2005	43	17/17/9/0	37/6	0.07 [0.01; 0.19]	0.67 [0.51; 0.81]
Caimmi	2006	Italy	Retrospective	2005–2006	2	1/1/0/0	2/0	0.00 [0.00; 0.84]	0.00 [0.00; 0.84]
Patane	2002	Italy	Retrospective	1991–1999	11	2/7/0/2	6/5	0.00 [0.00; 0.28]	0.00 [0.00; 0.28]
Koksal	2002	Turkey	Retrospective	2001–2002	2	0/2/0/0	2/0	0.00 [0.00; 0.84]	0.00 [0.00; 0.84]
Morishita	2001	Japan	Retrospective	1986–2000	6	3/2/1/0	4/2	0.00 [0.00; 0.46]	0.83 [0.36; 1.00]
Mariani	2001	Netherlands	Retrospective	1999–2000	3	2/1/0/0	3/0	0.00 [0.00; 0.71]	0.00 [0.00; 0.71]
Danton	1998	UK	Retrospective	1990–1997	13	6/3/2/2	11/2	0.00 [0.00; 0.25]	0.62 [0.32; 0.86]
Voets	1997	Netherlands	Case–control	1988–1995	24	8/14/2/0	24/0	0.21 [0.07; 0.42]	0.79 [0.58; 0.93]
Rao	1996	Canada	Retrospective	1982–1995	30	10/5/6/9	24/6	0.07 [0.01; 0.22]	0.23 [0.10; 0.42]
Francesca	1995	USA	Retrospective	1973–1990	21	11/7/3/0	18/3	0.05 [0.00; 0.24]	0.29 [0.11; 0.52]
Brutel	1995	Netherlands	Retrospective	1979–1993	79	22/48/9/0	69/10	0.06 [0.02; 0.14]	0.86 [0.76; 0.93]
Terzi	1994	Italy	Retrospective	1980–1993	10	2/3/3/2	10/0	0.10 [0.00; 0.45]	0.40 [0.12; 0.74]
Miller	1994	USA	Case–control	1965–1992	30	19/8/3/0	23/7	0.07 [0.01; 0.22]	0.50 [0.31; 0.69]
Yokoyama	1993	USA	Retrospective	1988–1992	11	6/4/1/0	9/2	0.00 [0.00; 0.28]	0.64 [0.31; 0.89]
Rosalion	1993	Australia	Retrospective	1987–1990	10	4/5/1/0	10/0	0.00 [0.00; 0.31]	0.80 [0.44; 0.97]
Ulicny	1992	USA	Retrospective	1980–1990	19	4/0/6/9	14/5	0.05 [0.00; 0.26]	1.00 [0.82; 1.00]
Canver	1990	USA	Retrospective	1982–1988	21	4/4/0/13	18/3	0.05 [0.00; 0.24]	0.10 [0.01; 0.30]

### Pooled proportion of operative mortality

**Fig. 2:**
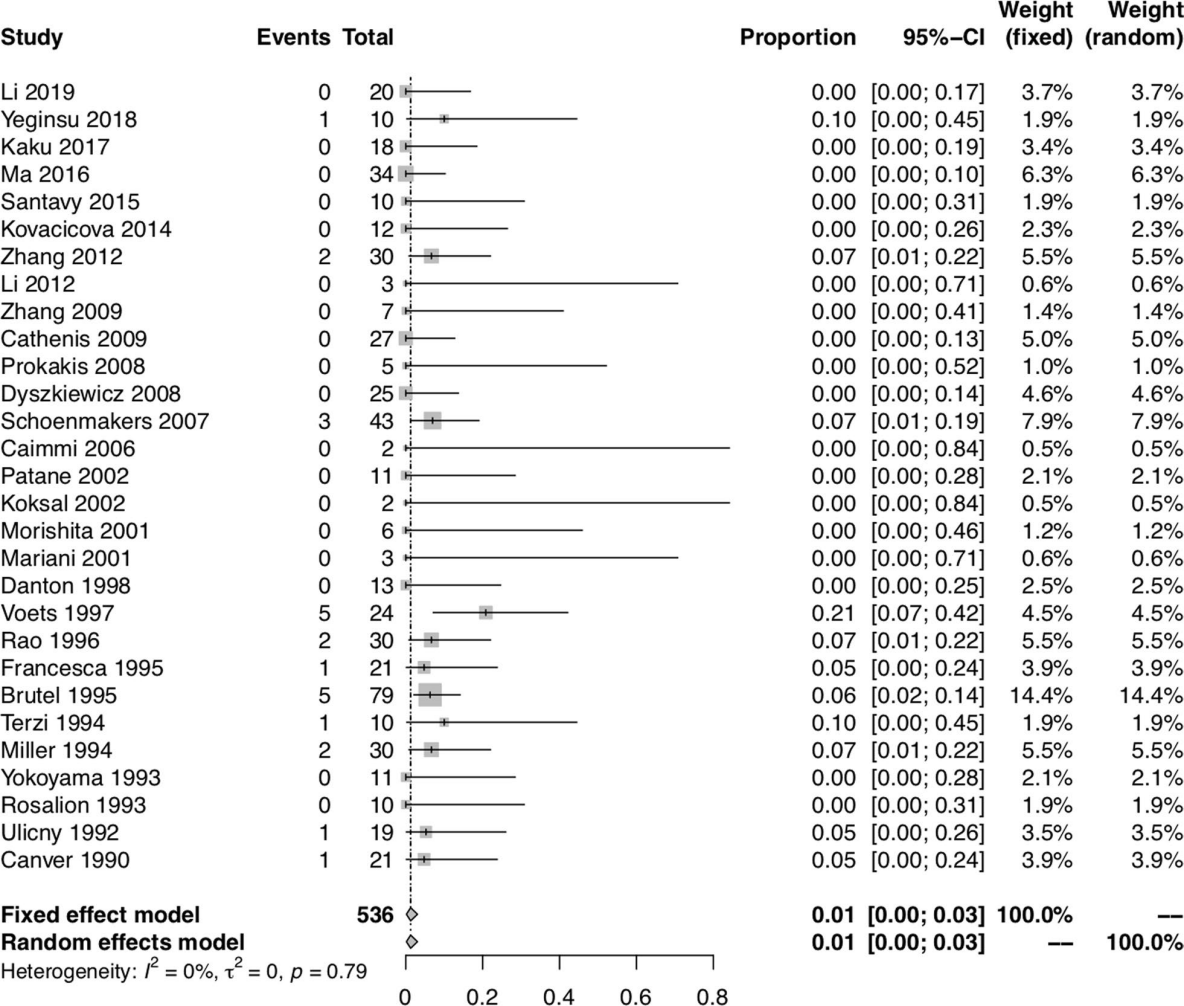
Forest plot of the pooled proportion of operative mortality in all studies

The pooled proportion of operative mortality was 0.01 (95% CI: 0.00, 0.03) using both fixed-effect and random-effect models. There was no significant heterogeneity between studies (I^2^ = 0.00%, *P* = 0.79) (Fig. 2).

### Pooled proportion of postoperative complications

**Fig. 3:**
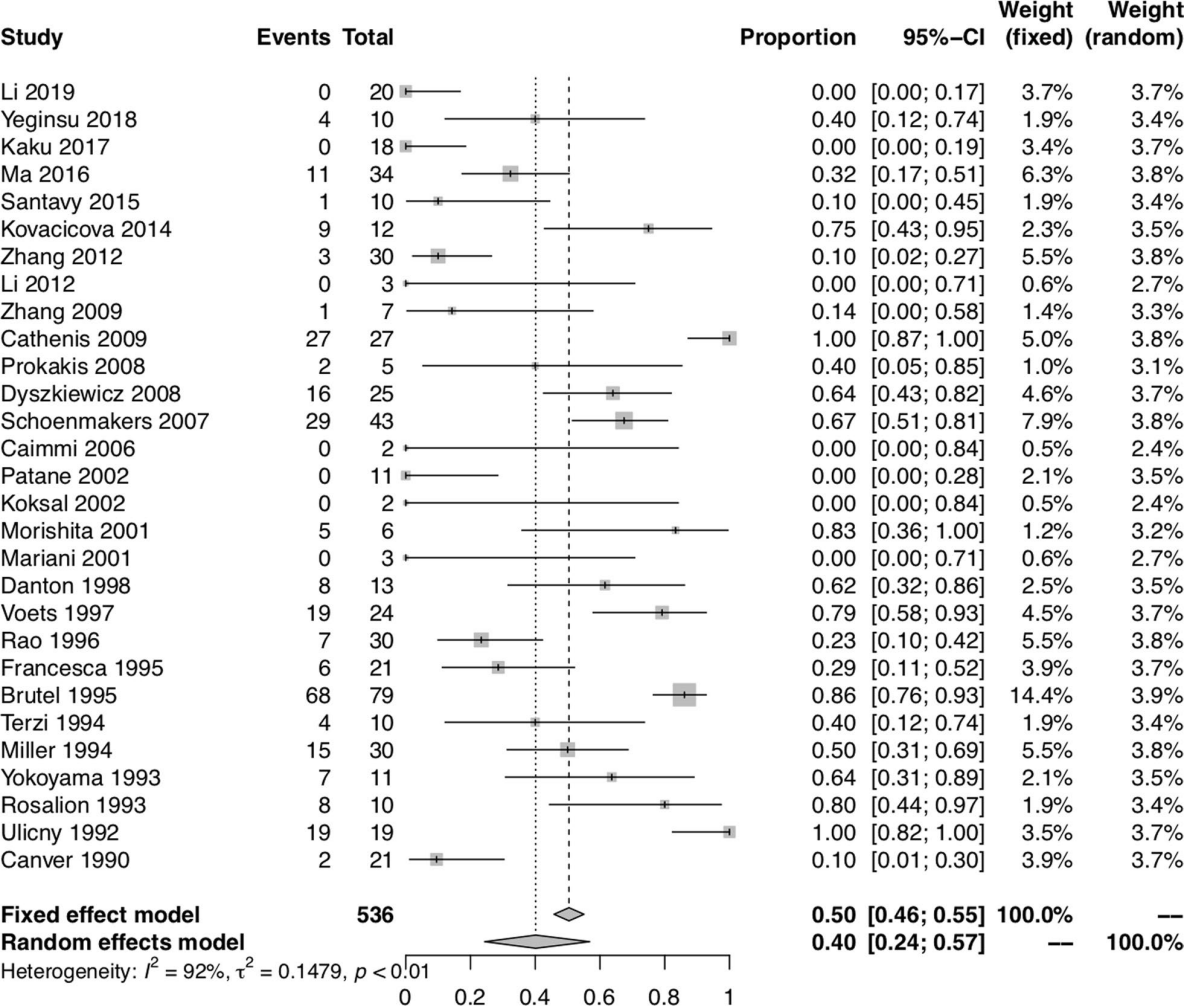
Forest plot of the pooled proportion of postoperative complications in all studies

**Fig. 4:**
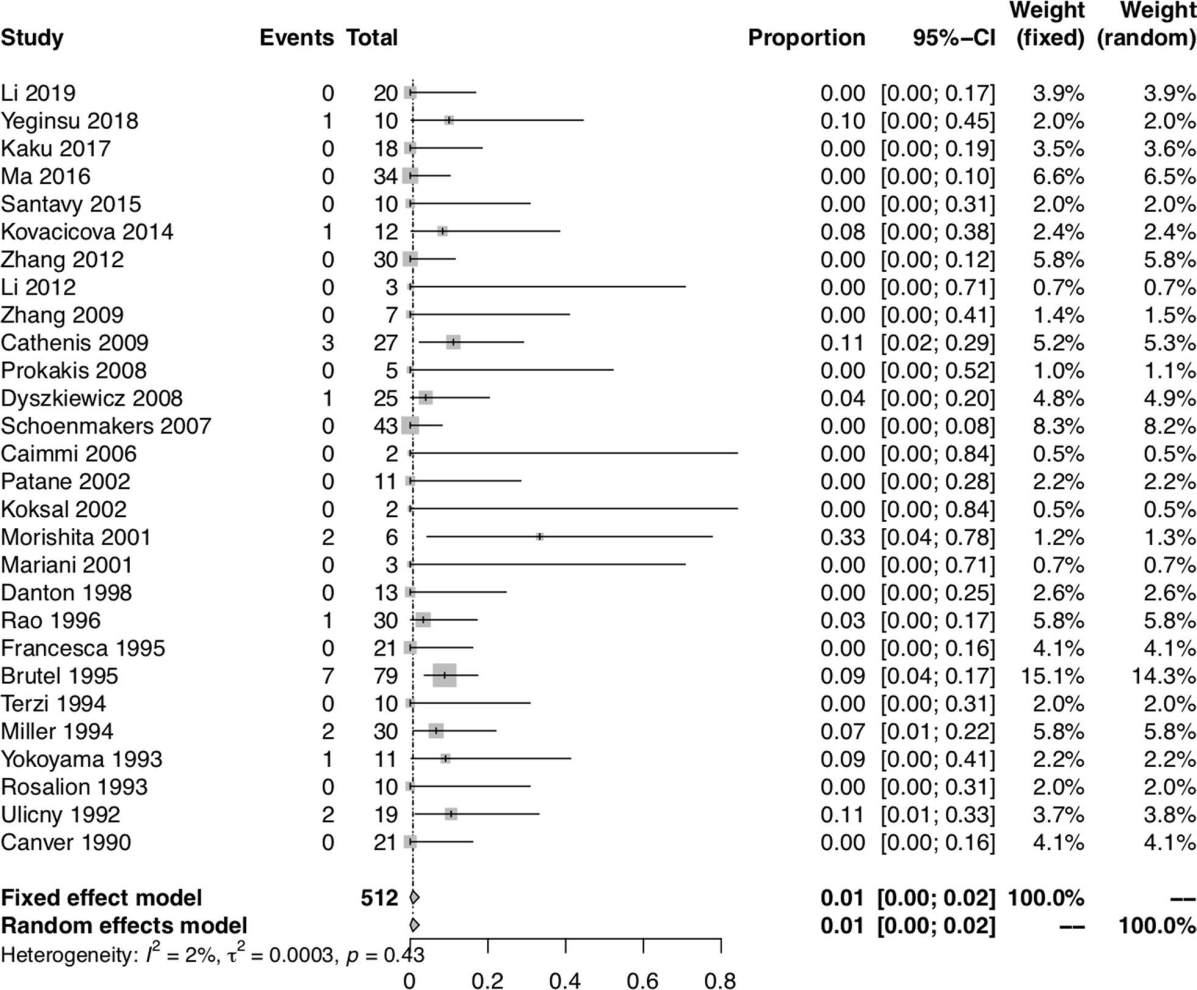
Forest plot of the pooled proportion of reopening for bleeding in all studies

The pooled proportion of postoperative complications was 0.40 (95% CI: 0.24, 0.57) using the random-effect model. There was significant heterogeneity between studies (I^2^ = 91.60%, *P* < 0.01) (Fig. 3). Furthermore, we divided the postoperative complications into three categories: reopening for bleeding; respiratory complications and cardiac complications. The pooled proportion of reopening for bleeding was 0.01 (95% CI: 0.00, 0.02) using both fixed-effect and random-effect models, with no significant heterogeneity between studies (I^2^ = 2.10%, *P* = 0.43) (Fig. 4). The pooled proportion of respiratory complications was 0.11 (95% CI: 0.04, 0.19) using the random-effect model, with significant heterogeneity between studies (I^2^ = 76.00%, *P* < 0.01). The pooled proportion of cardiac complications was 0.15 (95% CI: 0.08, 0.23) using the random-effect model, with significant heterogeneity between studies (I^2^ = 72.00%, *P* < 0.01).

### Subgroup analyses

To further assess the incidence rates of operative mortality and postoperative complications in patients who received combined heart surgery and lung tumor resection, we classified all patients into subgroups based on lung pathology and heart surgery procedures.

In the subgroups classified by lung pathology, 16 studies[3, 7–9, 11, 13–16, 19, 26, 28, 30, 32–34] contained 327 patients who received combined heart surgery and lung cancer resection, while the other 13 studies [5, 6, 10, 12, 17, 18, 20, 24, 25, 27, 29, 31, 35] enrolled patients with both malignant and benign lung diseases. Because malignant and benign lung diseases were analyzed together in these 13 studies [5, 6, 10, 12, 17, 18, 20, 24, 25, 27, 29, 31, 35], further assessment of benign diseases was not done. Therefore, we quantitatively pooled the data of 327 patients with lung cancer from the 16 studies [3, 7–9, 11, 13–16, 19, 26, 28, 30, 32–34]. The pooled proportion of operative mortality of patients who received combined heart surgery and lung cancer resection was 0.01 (95% CI: 0.00, 0.04) using both fixed-effect and random-effect models, with no significant heterogeneity between studies (I^2^ = 2.20%, *P* = 0.43). The pooled proportion of postoperative complications of patients who received combined heart surgery and lung cancer resection was 0.45 (95% CI: 0.27, 0.64) using the random-effect model, with significant heterogeneity between studies (I^2^ = 87.80%, *P* < 0.01).

**Fig. 5:**
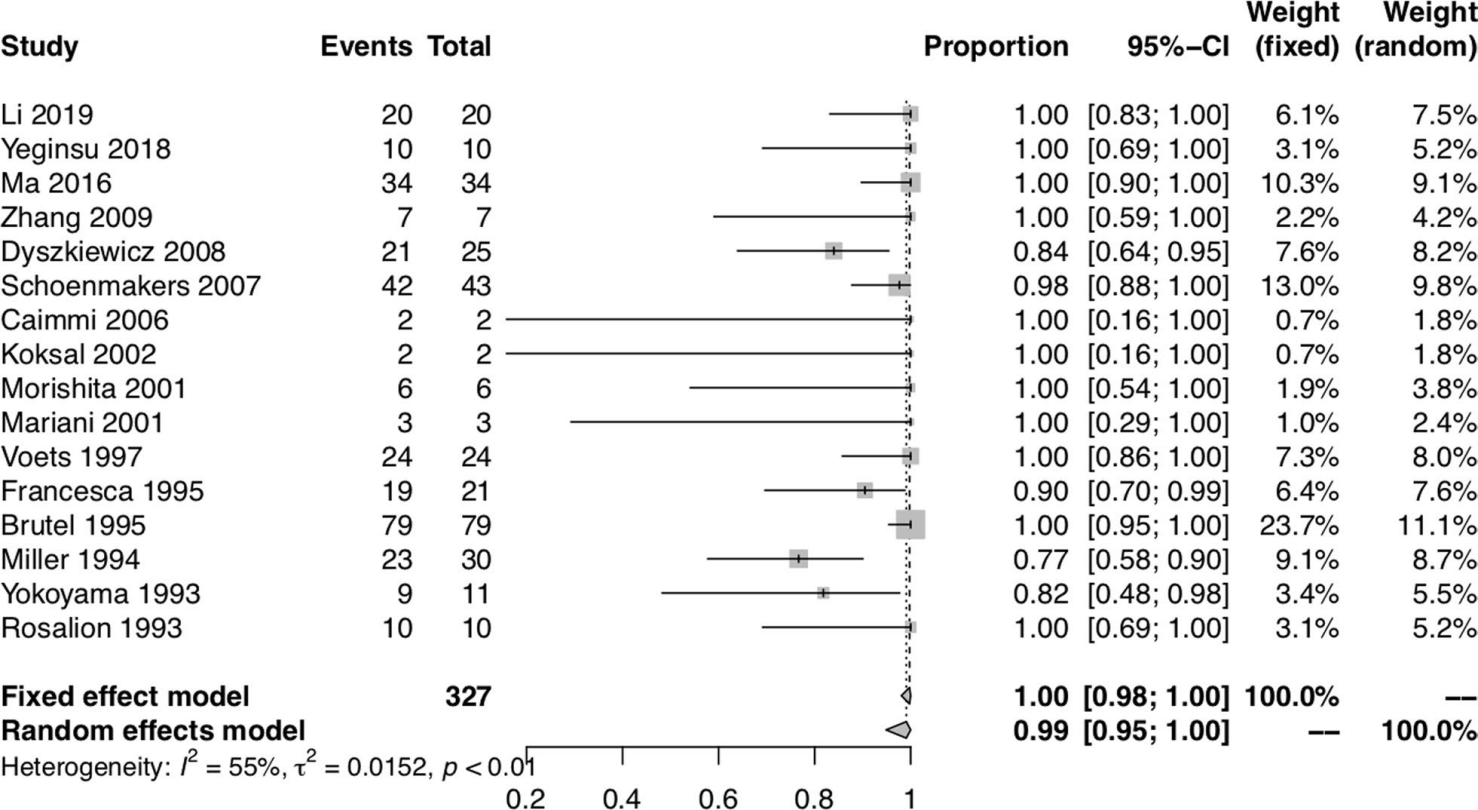
Forest plot of the pooled proportion of patients who underwent combined heart surgery and anatomical lung resection for lung cancer

**Fig. 6:**
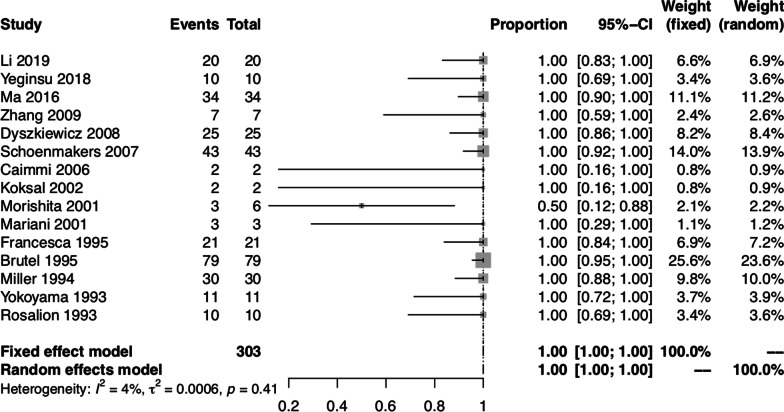
Forest plot of the pooled proportion of patients who underwent systematic lymph node dissection or sampling during combined heart surgery and lung cancer resection

To further evaluate the quality control of lung cancer surgery, we investigated whether patients with lung cancer underwent anatomical resection and systematic lymph node dissection or sampling. The pooled proportion of patients with lung cancer who underwent anatomical resection was 0.99 (95% CI: 0.95, 1.00) using the random-effect model, with significant heterogeneity between studies (I^2^ = 54.70%, *P* < 0.01) (Fig. 5). Among the 16 studies that only included patients with malignant lung lesions [3, 7–9, 11, 13–16, 19, 26, 28, 30, 32–34], 1 study did not provide enough information about lymph node dissection or sampling [7]. Therefore, 15 studies [3, 8, 9, 11, 13–16, 19, 26, 28, 30, 32–34] containing 303 patients who received combined heart surgery and lung cancer resection were used to analyze the proportion of systematic lymph node dissection or sampling. The pooled proportion of patients with lung cancer who underwent systematic lymph node dissection or sampling was 1.00 (95% CI: 1.00, 1.00) using both fixed-effect and random-effect models, with no significant heterogeneity between studies (I^2^ = 4.20%, *P* = 0.41) (Fig. 6).

In the subgroups classified by heart surgery procedures, 10 studies [3, 9, 11, 14, 15, 18, 26, 28, 30, 34] contained 123 patients who received combined coronary artery bypass grafting (CABG) surgery and lung tumor resection, while the other 19 studies [5–8, 10, 12, 13, 16, 17, 19, 20, 24, 25, 27, 29, 31–33, 35] enrolled patients who underwent either CABG surgery or other types of heart surgery procedures. Because CABG surgery and other types of heart surgery procedures were analyzed together in these 19 studies [5–8, 10, 12, 13, 16, 17, 19, 20, 24, 25, 27, 29, 31–33, 35], further assessment of heart surgery procedures other than CABG was not done. Therefore, we quantitatively pooled the data of 123 patients who underwent CABG in 10 studies [3, 9, 11, 14, 15, 18, 26, 28, 30, 34]. The pooled proportion of operative mortality of patients who received combined CABG surgery and lung tumor resection was 0.00 (95% CI: 0.00, 0.01) using both fixed-effect and random-effect models, with no significant heterogeneity between studies (I^2^ = 0.00%, *P* = 0.76). The pooled proportion of postoperative complications of patients who received combined CABG surgery and lung tumor resection was 0.26 (95% CI: 0.07, 0.50) using the random-effect model, with significant heterogeneity between studies (I^2^ = 79.80%, *P* < 0.01).

**Fig. 7:**
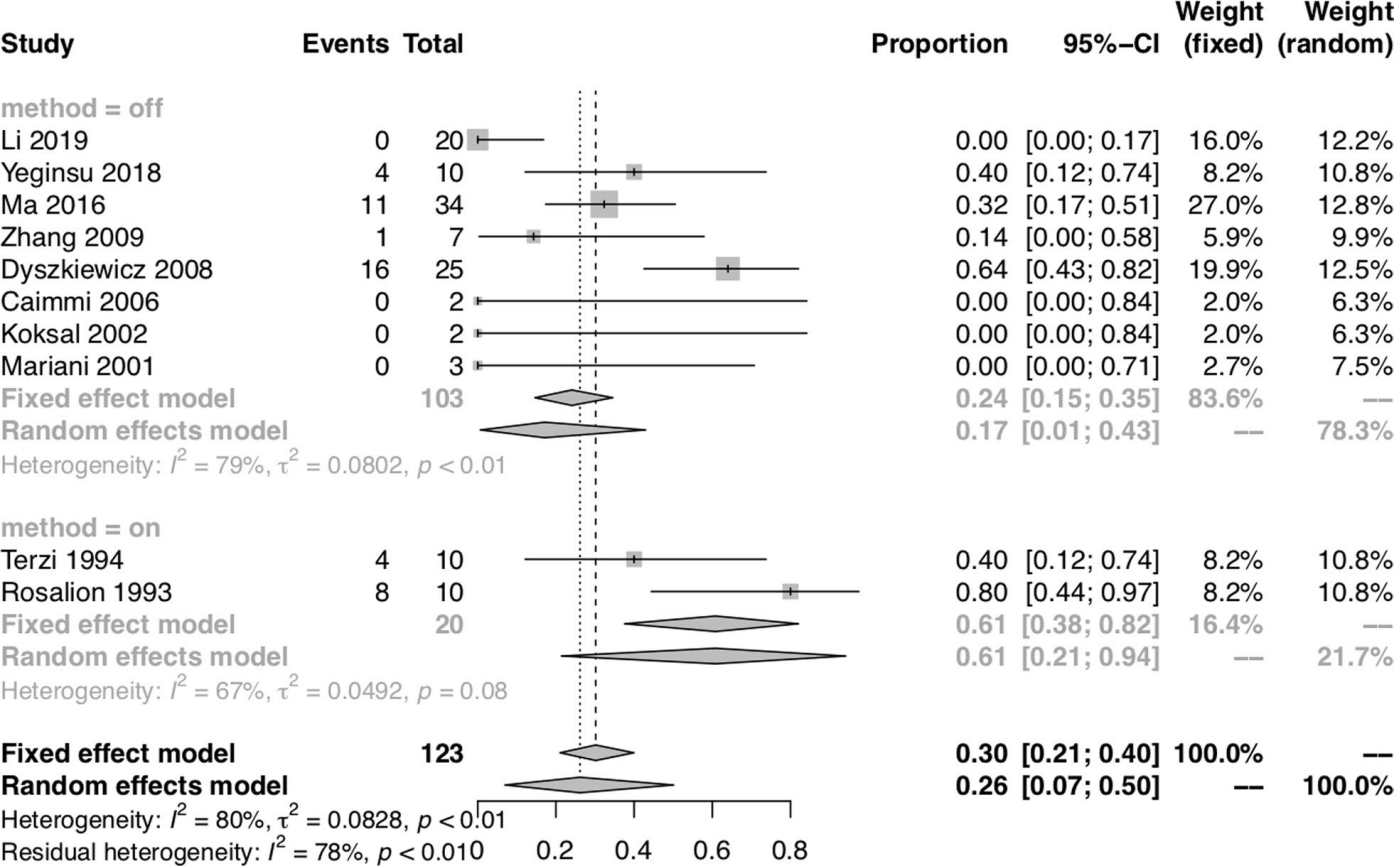
Forest plot of pooled studies for the association between off-pump or on-pump method and postoperative complications rates in patients who received combined CABG surgery and lung tumor resection

To further evaluate the impact of CPB on postoperative complications, we compared the effect of the off-pump versus the on-pump method on postoperative complications in patients who underwent CABG. The pooled proportion of postoperative complications of patients who underwent CABG using the off-pump method was 0.17 (95% CI: 0.01, 0.43) using the random-effect model, with significant heterogeneity between studies (I^2^ = 79.40%, *P* < 0.01). The pooled proportion of postoperative complications of patients who underwent CABG using the on-pump method was 0.61 (95% CI: 0.38, 0.82) using the fixed-effect model, with no significant heterogeneity between studies (I^2^ = 67.40%, *P* = 0.08) (Fig. 7).

### Sensitivity analysis and publication bias

**Fig. 8:**
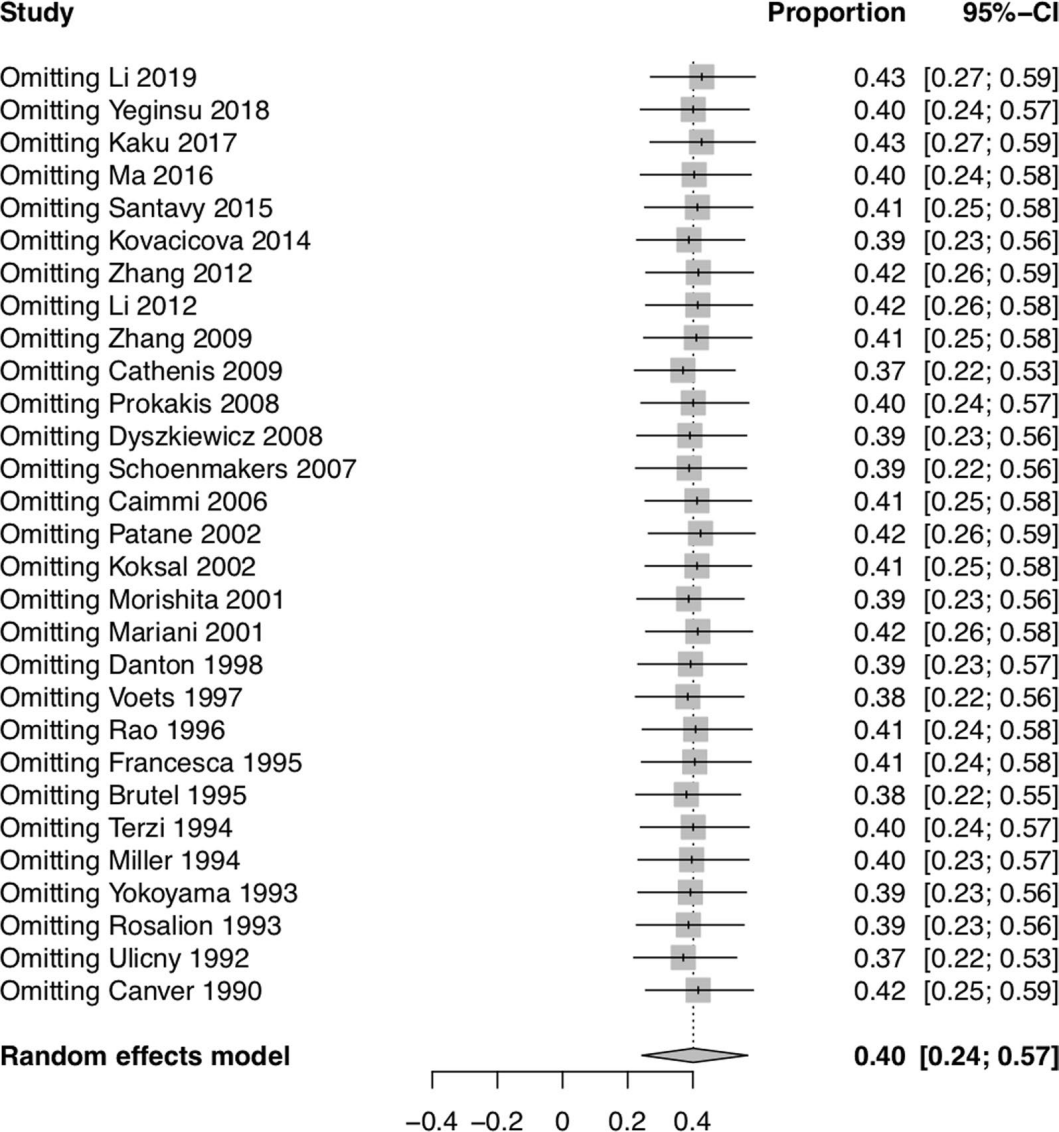
Sensitivity analysis for the pooled proportion of postoperative complications for all studies

**Fig. 9:**
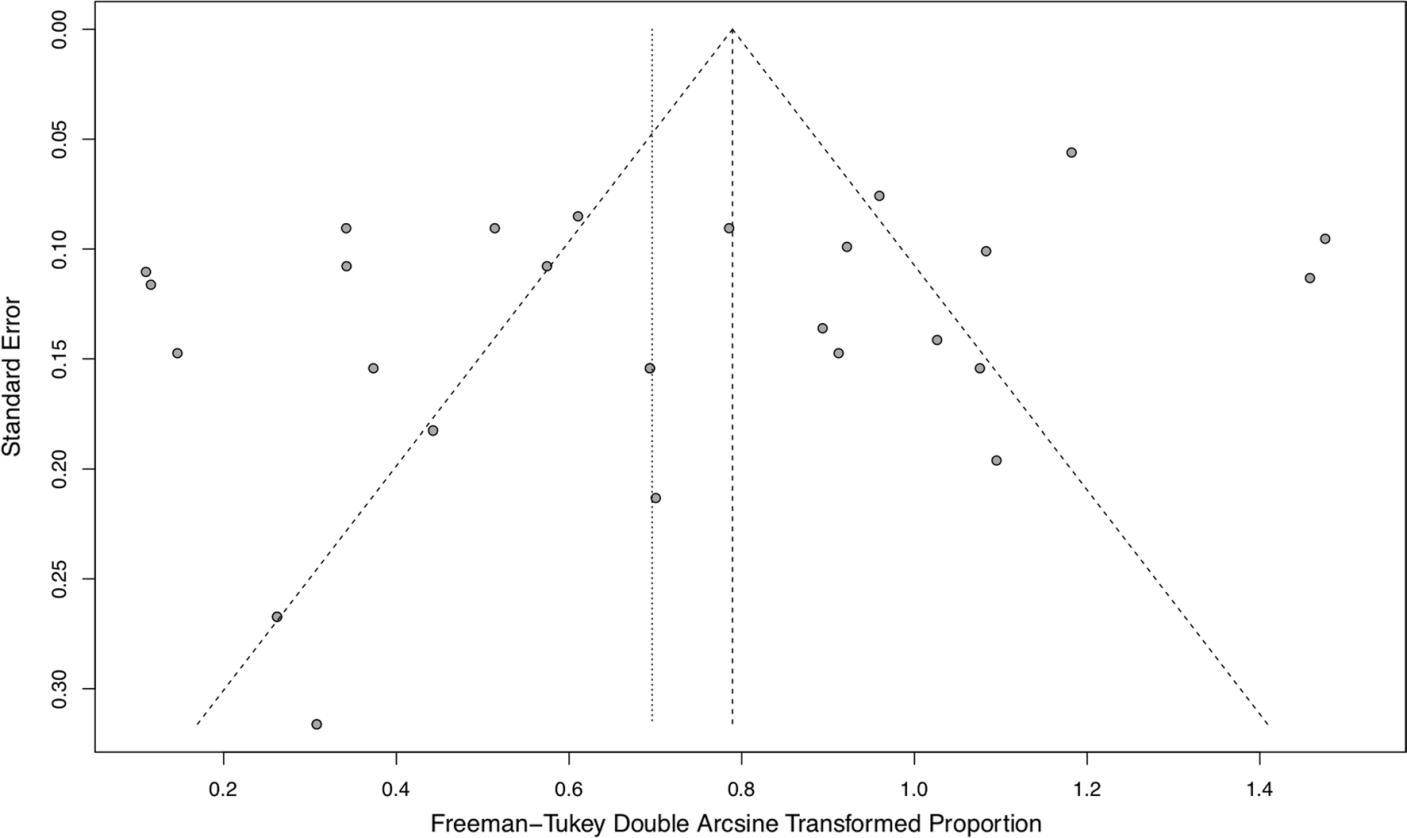
Funnel plot for publication bias of the pooled proportion of postoperative complications for all studies

Sensitivity analysis showed that no individual study influenced the overall results (Fig. 8). Funnel plot for publication bias of the pooled proportion of postoperative complications for all studies was shown in Fig. 9. There was no significant publication bias detected using Begg’s test (*P* = 0.42) and Egger’s test (*P* = 0.07).

## Discussion

This systematic review and meta-analysis involved a total of 536 patients who received combined heart surgery and lung tumor resection in 29 observational studies. Our comprehensive data analysis demonstrated that combined heart surgery and lung tumor resection had a low mortality rate and an acceptable complication rate. The incidence rate of reopening for bleeding was quite low. The incidence rates of postoperative respiratory complications and cardiac complications were similar. Overall, the present results suggest that combined heart surgery and lung tumor resection is safe with low mortality and complication rates.

A previous systematic review by Tourmousoglou and associates included 15 retrospective studies from 1994 to 2012 [36]. They compared the operative mortality rate, reoperation rate, and survival rate between combined and staged surgery for patients with both coronary artery disease and lung cancer. Without quantitative comparison, the authors found that the operative mortality rate was 0–20.8% for combined procedures and 0–10% for staged procedures. The reoperation rate for bleeding was 0–11% for combined procedures and 0% for staged procedures. Similar to our results, a more recent systematic review and meta-analysis by Bablekos and co-workers included 22 retrospective studies from 1985 to 2011 [37]. They summarized the operative mortality rate, complication rate, and survival rate of lung cancer patients complicated with heart disease. Without performing subgroup analysis of simultaneous or staged surgery, they found the pooled operative mortality rate was 5.26% (95% CI: 3.47, 7.62) and the pooled complication rate was 45.59% (95% CI: 35.62, 55.74).

Patients who undergo combined heart surgery and lung tumor resection face a potential risk of postoperative bleeding. This concern raises from CPB use during heart surgery and, while performing heart and lung surgery simultaneously, the more complicated surgical procedure. Potential bleeding might be related to excessive heparin use, inadequate heparin neutralization, or platelet dysfunction during CPB [38, 39]. Bleeding might arise from the area of the lung resection, extensive mediastinal lymph node dissection and, not less frequently, intrapulmonary hemorrhage [20, 40]. Excessive bleeding increases the reexploration rate and eventually increases the mortality and complication rates. Off-pump CABG is one solution to reduce the potential risk of postoperative bleeding. During off-pump CABG, the intraoperative heparin dose was reduced and the platelet functions were less affected, leading to decreased bleeding [41]. Alternatively, when CPB was necessary, the lung resection and lymph node dissection were best performed before heparinization or after reversal of heparin by protamine sulfate [19, 35]. Furthermore, compared with wedge excisions, anatomical lung resection was preferred to reduce the risk of intrapulmonary hemorrhage [20, 40].

In the subgroups classified by lung pathology, we focused on patients who underwent combined heart surgery and lung cancer resection. As median sternotomy was often employed as a single incision to perform simultaneous cardiac and pulmonary surgery, there were concerns regarding the safety and adequacy of oncological pulmonary and lymph node resection [6, 8, 14, 17]. Through a median sternotomy, left lower lobectomy was considered the most technically difficult pulmonary lobectomy [9, 12, 16, 33, 42]. The intraoperative view of left lower lobectomy was obscured by the heart. Retraction of the heart was often needed, but possibly led to arrhythmias and hemodynamic instability. To facilitate exposure of the left lower lobe, CPB could be used to reduce the heart volume. Also, adding a lateral thoracotomy to the median sternotomy or extending the median sternotomy into the intercostal space could be adopted. Compared with a lateral thoracotomy, performing mediastinal lymph node dissection through a median sternotomy was more technically difficult and time-consuming. Although systematic mediastinal lymph node dissection through a median sternotomy was possible, the subcarinal and posterior mediastinal lymph nodes were particularly difficult to sample [8, 31]. One solution was to open the posterior pericardium and mobilize the right pulmonary artery, allowing a complete dissection of the subcarinal space. Also, adding a lateral thoracotomy to the median sternotomy could be adopted.

In the subgroups classified by heart surgery procedures, we focused on patients who underwent combined CABG surgery and lung tumor resection. We compared the effect of off-pump versus on-pump CABG on postoperative complications and found that off-pump CABG could reduce the complication rate. Many disadvantages of CPB have been described. With higher doses of heparin, CPB may increase the risk of bleeding [39]. In addition, CPB circuits manufactured from synthetic materials may impair platelet function and enhance systemic inflammatory response syndrome [43]. Furthermore, CPB may cause edema of the peribronchial and lung tissue, as well as lung injury [44–46], and may impair the immune system and promote tumor cell metastasis [47]. After eliminating the shortcomings of CPB, off-pump CABG might reduce the complication rate.

The present meta-analysis has several limitations that should be acknowledged. First, there was significant heterogeneity between included studies, which was likely due to clinical and methodological differences between studies. Second, all included studies were retrospective observational studies. Third, survival data were not evaluated in our study. Our study focused on perioperative outcomes such as the incidence rates of operative mortality and postoperative complications. Fourth, articles comparing combined and staged surgery were so limited. Only three articles were found from our comprehensive literature search. So, the comparison of mortality and complication rates of combined cardiothoracic surgery and staged surgery was not performed in our study. More studies are needed to compare the mortality and morbidity of combined and staged surgery. Fifth, none of the included studies had described in detail whether complete or incomplete revascularization was achieved in coronary artery disease patients. So, the comparison between complete and incomplete revascularization for coronary artery disease patients was not performed in our study.

## Conclusion

Our systematic meta-analysis demonstrated that combined heart surgery and lung tumor resection had a low mortality rate and an acceptable complication rate. According to the subgroup analysis, although the procedures were technically difficult and time-consuming, most patients with lung cancer successfully underwent anatomical resection and mediastinal lymph node sampling or dissection. For patients receiving combined CABG surgery and lung tumor resection, off-pump CABG may potentially reduce the complication rate compared with on-pump CABG. Considering the limitations of this study, our findings need to be further confirmed in large-scale prospective research.

## Data Availability

All data generated or analyzed during this study are included in this published article.

## Abbreviations

CABG: Coronary artery bypass grafting
CPB: Cardiopulmonary bypass
NOS: Newcastle–Ottawa Scale
